# 2-(3-Oxo-3,4-dihydro-2*H*-1,4-benzothia­zin-4-yl)acetohydrazide

**DOI:** 10.1107/S1600536810031272

**Published:** 2010-08-11

**Authors:** Azher Saeed, Zaid Mahmood, Shiyao Yang, Saeed Ahmad, Muhammad Salim

**Affiliations:** a Institute of Chemistry, University of the Punjab, Lahore 54590, Pakistan; b Department of Chemistry, College of Chemistry and Chemical Engineering, Xiamen University, Xiamen 361005, People’s Republic of China; c Department of Chemistry, Gomal University, Dera Ismaeel Khan, Pakistan

## Abstract

In the title compound, C_10_H_11_N_3_O_2_S, the thia­zine ring exists in a conformation inter­mediate between twist-boat and half-chair. The dihedral angle between the mean plane of the thia­zine ring and the hydrazide group is 89.45 (13)°. In the crystal, N—H⋯O hydrogen bonds link the mol­ecules into (100) sheets and weak C—H⋯O inter­actions further consolidate the packing.

## Related literature

For the biological and medicinal activity of 1,4-benzothia­zine compounds, see: Armenise *et al.* (1991 ▶); Gupta *et al.* (1993 ▶); Vicente *et al.* (2009 ▶); Schiaffella *et al.* (2006 ▶); Kaneko *et al.* (2002 ▶). For the pharmacological properties of hydrazones and their derivatives, see: Sivasankar *et al.* (1995 ▶); Satyanarayana *et al.* (2008 ▶). For hydrogen-bond motifs, see: Bernstein *et al.* (1995 ▶).
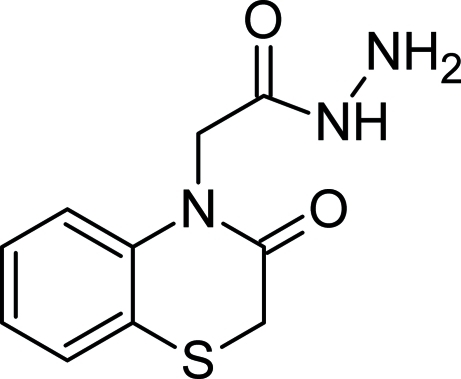


## Experimental

### 

#### Crystal data

C_10_H_11_N_3_O_2_S
*M* *_r_* = 237.28
Monoclinic, 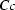

*a* = 15.3744 (10) Å
*b* = 7.5162 (5) Å
*c* = 9.6256 (7) Å
β = 95.413 (3)°
*V* = 1107.35 (13) Å^3^
*Z* = 4
Mo *K*α radiation
μ = 0.28 mm^−1^
*T* = 296 K
0.46 × 0.23 × 0.20 mm

#### Data collection

Bruker Kappa APEXII CCD diffractometer
Absorption correction: multi-scan (*SADABS*; Bruker, 2007 ▶) *T*_min_ = 0.882, *T*_max_ = 0.946
6103 measured reflections
2168 independent reflections
2077 reflections with *I* > 2σ(*I*)
*R*_int_ = 0.021

#### Refinement

*R*[*F* ^2^ > 2σ(*F* ^2^)] = 0.027
*wR*(*F* ^2^) = 0.076
*S* = 1.04
2168 reflections
157 parameters
2 restraints
H atoms treated by a mixture of independent and constrained refinement
Δρ_max_ = 0.18 e Å^−3^
Δρ_min_ = −0.14 e Å^−3^
Absolute structure: Flack (1983 ▶), 792 Friedel pairs
Flack parameter: 0.00 (7)

### 

Data collection: *APEX2* (Bruker, 2007 ▶); cell refinement: *SAINT* (Bruker, 2007 ▶); data reduction: *SAINT*; program(s) used to solve structure: *SHELXS97* (Sheldrick, 2008 ▶); program(s) used to refine structure: *SHELXL97* (Sheldrick, 2008 ▶); molecular graphics: *ORTEP-3* (Farrugia, 1997 ▶) and *PLATON* (Spek, 2009 ▶); software used to prepare material for publication: *WinGX* (Farrugia, 1999 ▶) and *PLATON*.

## Supplementary Material

Crystal structure: contains datablocks I, global. DOI: 10.1107/S1600536810031272/hb5598sup1.cif

Structure factors: contains datablocks I. DOI: 10.1107/S1600536810031272/hb5598Isup2.hkl

Additional supplementary materials:  crystallographic information; 3D view; checkCIF report

## Figures and Tables

**Table 1:** Hydrogen-bond geometry (Å, °)

*D*—H⋯*A*	*D*—H	H⋯*A*	*D*⋯*A*	*D*—H⋯*A*
N3—H3*N*⋯O2^i^	0.96 (3)	2.35 (3)	3.299 (3)	171 (2)
N2—H1*N*⋯O1^ii^	0.87 (2)	2.07 (2)	2.935 (2)	175.5 (18)
C3—H3⋯O2^iii^	0.93	2.48	3.406 (3)	174
C8—H8*B*⋯O1^iv^	0.97	2.57	3.442 (2)	150

Symmetry codes: (i) 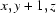; (ii) 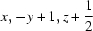; (iii) 
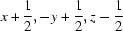
; (iv) 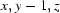.

**Fig. 1.**
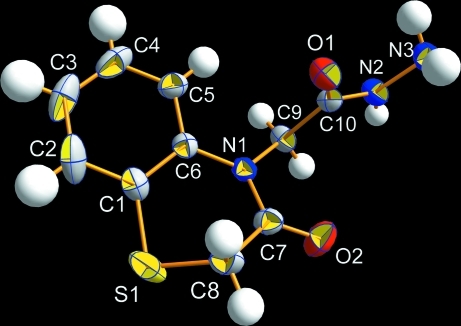
The molecular structure of the title compound, with displacement ellipsoids drawn at the 50% probability level.

**Fig. 2.**
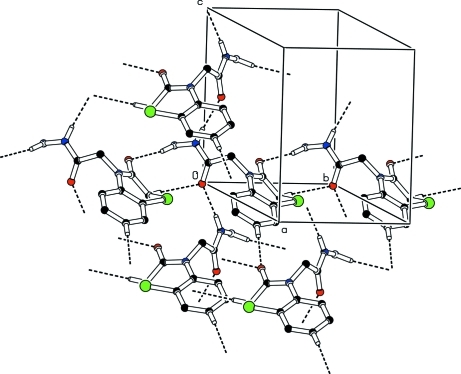
The crystal packing of the title compound and Intermolecular hydrogen bonds are shown by dashed lines.

## supplementary crystallographic information

### Comment

The 4H-benzo(1,4)thiazine compounds exhibit a broad spectrum of biological activitives, such as tetramic acids substituted benzothiazine derivatives are potent inhibitors of HCV polymerase (Vicente et al., 2009)and the pyrazino subsituted 1,4-benzothiazine derivatives are inhibitors of adhesion molecule-1, (Kaneko et al., 2002).They are also known to have antibacterial (Armenise  et al.,1991),anticancer (Gupta et al.,1993),  antifungal (Schiaffella et al., 2006) activities. The hydrazone compounds were known for their coordinating capability, pharmacological activity, antibacterial and antifungal properties (Sivasankar et al., 1995) (Satyanarayana, et al., 2008). We  paid attention to the preparation of hydrazone derivatives of 2-(3-oxo-2,3-dihydro-2H-1,4-benzothiazin-3-one and we report here the structure of the title compound.

The dihedral angle between the aromatic benzene ring C1–C6 and thiazine ring C1/C6/N1/C7/C8/S1 is 16.77(0.10)°  while the hydrazide group C9/C10/N2/N3 is oriented at dihedral angle of 89.45(0.13)° with respect to the thiazine ring. The symmetry related intermolecular N—H···O and C—H···O interaction form the dimer along the b axis which results in a ring motif R_2_^2^(9) (Bernstein et al., 1995). The crystal structure is further stabilized through the N—H···O and week C—H···O interaction to form three dimentional network.

### Experimental

Ethyl 2-(3-oxo-2,3-dihydrobenzo[b][1,4]thiazin-4-yl)acetate (1.26 g, 5mmol) was refluxed in 50 ml ethanol with 2.0 ml of hydrazine for 5 hours. On completion the solution was evaporated under reduce pressure and solid obtained was purified from ethanol.  Colourless needles of (I) were obtained by slow evaporation from methanol (m.p. 430 K).

### Refinement

The C-H H-atoms were positioned with idealized geometry with C—H = 0.93 Å and were refined using a riding model with *U*_iso_(H) = 1.2 *U*_eq_(C). The N-H H atoms were located in difference map with N—H= 0.76 (4)—0.83 (4) Å, *U*_iso_(H) = 1.2 for N atoms.

### Crystal data

**Table:**

C_10_H_11_N_3_O_2_S	*F*(000) = 496
*M**_r_* = 237.28	*D*_x_ = 1.423 Mg m^−^^3^
Monoclinic, *C**c*	Melting point: 430 K
Hall symbol: C -2yc	Mo *K*α radiation, λ = 0.71073 Å
*a* = 15.3744 (10) Å	Cell parameters from 4206 reflections
*b* = 7.5162 (5) Å	θ = 3.0–31.2°
*c* = 9.6256 (7) Å	µ = 0.28 mm^−^^1^
β = 95.413 (3)°	*T* = 296 K
*V* = 1107.35 (13) Å^3^	Needle, colorless
*Z* = 4	0.46 × 0.23 × 0.20 mm

### Data collection

**Table:**

Bruker Kappa APEXII CCD diffractometer	2168 independent reflections
Radiation source: fine-focus sealed tube	2077 reflections with *I* > 2σ(*I*)
graphite	*R*_int_ = 0.021
φ and ω scans	θ_max_ = 28.4°, θ_min_ = 2.7°
Absorption correction: multi-scan (*SADABS*; Bruker, 2007)	*h* = −12→20
*T*_min_ = 0.882, *T*_max_ = 0.946	*k* = −8→10
6103 measured reflections	*l* = −12→10

### Refinement

**Table:**

Refinement on *F*^2^	Secondary atom site location: difference Fourier map
Least-squares matrix: full	Hydrogen site location: inferred from neighbouring sites
*R*[*F*^2^ > 2σ(*F*^2^)] = 0.027	H atoms treated by a mixture of independent and constrained refinement
*wR*(*F*^2^) = 0.076	*w* = 1/[σ^2^(*F*_o_^2^) + (0.0437*P*)^2^ + 0.2667*P*] where *P* = (*F*_o_^2^ + 2*F*_c_^2^)/3
*S* = 1.04	(Δ/σ)_max_ < 0.001
2168 reflections	Δρ_max_ = 0.18 e Å^−^^3^
157 parameters	Δρ_min_ = −0.14 e Å^−^^3^
2 restraints	Absolute structure: Flack (1983), 792 Friedel pairs
Primary atom site location: structure-invariant direct methods	Flack parameter: 0.00 (7)

### Special details

**Table:**

Geometry. All esds (except the esd in the dihedral angle between two l.s. planes) are estimated using the full covariance matrix. The cell esds are taken into account individually in the estimation of esds in distances, angles and torsion angles; correlations between esds in cell parameters are only used when they are defined by crystal symmetry. An approximate (isotropic) treatment of cell esds is used for estimating esds involving l.s. planes.
Refinement. Refinement of F^2^ against ALL reflections. The weighted R-factor wR and goodness of fit S are based on F^2^, conventional R-factors R are based on F, with F set to zero for negative F^2^. The threshold expression of F^2^ > 2sigma(F^2^) is used only for calculating R-factors(gt) etc. and is not relevant to the choice of reflections for refinement. R-factors based on F^2^ are statistically about twice as large as those based on F, and R- factors based on ALL data will be even larger.

### Fractional atomic coordinates and isotropic or equivalent isotropic displacement parameters (Å^2^)

**Table:**

	*x*	*y*	*z*	*U*_iso_*/*U*_eq_	
C1	0.81283 (13)	0.1402 (3)	0.4407 (2)	0.0444 (4)	
C2	0.88393 (15)	0.1642 (4)	0.3624 (3)	0.0684 (7)	
H2	0.9061	0.0674	0.3170	0.082*	
C3	0.92138 (15)	0.3276 (5)	0.3514 (3)	0.0727 (7)	
H3	0.9691	0.3412	0.2998	0.087*	
C4	0.88855 (14)	0.4717 (4)	0.4166 (3)	0.0621 (6)	
H4	0.9145	0.5827	0.4099	0.074*	
C5	0.81688 (12)	0.4524 (2)	0.4924 (2)	0.0451 (4)	
H5	0.7944	0.5514	0.5347	0.054*	
C6	0.77806 (10)	0.2868 (2)	0.50596 (15)	0.0325 (3)	
C7	0.64582 (13)	0.1320 (2)	0.56774 (18)	0.0393 (4)	
C8	0.65696 (13)	−0.0005 (2)	0.4532 (2)	0.0473 (4)	
H8A	0.6398	0.0537	0.3633	0.057*	
H8B	0.6194	−0.1024	0.4638	0.057*	
C9	0.68864 (12)	0.3985 (2)	0.69169 (16)	0.0371 (4)	
H9A	0.7419	0.4638	0.7190	0.044*	
H9B	0.6710	0.3388	0.7738	0.044*	
C10	0.61760 (11)	0.52828 (19)	0.63748 (15)	0.0311 (3)	
N1	0.70588 (9)	0.26545 (17)	0.58681 (13)	0.0328 (3)	
N2	0.57596 (11)	0.60513 (19)	0.73773 (16)	0.0391 (3)	
N3	0.50598 (12)	0.7263 (3)	0.70912 (19)	0.0491 (4)	
O1	0.60169 (9)	0.56431 (16)	0.51324 (12)	0.0441 (3)	
O2	0.58443 (11)	0.1213 (2)	0.63853 (16)	0.0602 (4)	
S1	0.76839 (4)	−0.07275 (6)	0.45907 (6)	0.06435 (19)	
H1N	0.5831 (14)	0.561 (3)	0.822 (3)	0.036 (5)*	
H2N	0.4722 (19)	0.678 (4)	0.641 (3)	0.072 (9)*	
H3N	0.5321 (18)	0.834 (4)	0.681 (3)	0.065 (8)*	

### Atomic displacement parameters (Å^2^)

**Table:**

	*U*^11^	*U*^22^	*U*^33^	*U*^12^	*U*^13^	*U*^23^
C1	0.0391 (9)	0.0492 (9)	0.0446 (10)	0.0071 (7)	0.0020 (8)	−0.0098 (8)
C2	0.0384 (11)	0.108 (2)	0.0598 (13)	0.0113 (12)	0.0099 (10)	−0.0256 (14)
C3	0.0360 (10)	0.126 (2)	0.0580 (14)	−0.0063 (14)	0.0133 (9)	0.0076 (15)
C4	0.0409 (10)	0.0773 (15)	0.0673 (14)	−0.0139 (10)	0.0019 (10)	0.0272 (12)
C5	0.0411 (10)	0.0407 (9)	0.0531 (12)	−0.0021 (8)	0.0022 (8)	0.0107 (8)
C6	0.0334 (8)	0.0347 (7)	0.0288 (8)	0.0042 (6)	0.0004 (6)	0.0033 (6)
C7	0.0462 (10)	0.0359 (8)	0.0360 (9)	−0.0013 (7)	0.0057 (7)	0.0027 (7)
C8	0.0532 (11)	0.0358 (9)	0.0529 (11)	−0.0081 (7)	0.0049 (8)	−0.0080 (7)
C9	0.0463 (9)	0.0382 (8)	0.0266 (8)	0.0068 (7)	0.0026 (7)	−0.0034 (6)
C10	0.0391 (8)	0.0292 (7)	0.0253 (7)	−0.0012 (6)	0.0052 (6)	−0.0015 (5)
N1	0.0393 (7)	0.0293 (6)	0.0304 (7)	0.0025 (5)	0.0068 (6)	−0.0012 (5)
N2	0.0516 (9)	0.0405 (7)	0.0261 (7)	0.0102 (6)	0.0081 (6)	0.0003 (6)
N3	0.0518 (10)	0.0517 (10)	0.0455 (9)	0.0147 (8)	0.0134 (8)	0.0016 (8)
O1	0.0585 (8)	0.0504 (7)	0.0239 (6)	0.0137 (6)	0.0059 (5)	0.0007 (5)
O2	0.0559 (9)	0.0728 (9)	0.0553 (9)	−0.0161 (7)	0.0223 (7)	−0.0008 (8)
S1	0.0661 (3)	0.0354 (2)	0.0898 (4)	0.0116 (2)	−0.0018 (3)	−0.0191 (3)

### Geometric parameters (Å, °)

**Table:**

C1—C2	1.397 (3)	C7—C8	1.508 (3)
C1—C6	1.400 (2)	C8—S1	1.793 (2)
C1—S1	1.756 (2)	C8—H8A	0.9700
C2—C3	1.364 (4)	C8—H8B	0.9700
C2—H2	0.9300	C9—N1	1.463 (2)
C3—C4	1.372 (4)	C9—C10	1.520 (2)
C3—H3	0.9300	C9—H9A	0.9700
C4—C5	1.385 (3)	C9—H9B	0.9700
C4—H4	0.9300	C10—O1	1.228 (2)
C5—C6	1.392 (2)	C10—N2	1.338 (2)
C5—H5	0.9300	N2—N3	1.417 (2)
C6—N1	1.423 (2)	N2—H1N	0.87 (2)
C7—O2	1.218 (2)	N3—H2N	0.87 (3)
C7—N1	1.364 (2)	N3—H3N	0.96 (3)
			
C2—C1—C6	119.5 (2)	S1—C8—H8A	109.5
C2—C1—S1	120.28 (17)	C7—C8—H8B	109.5
C6—C1—S1	120.24 (15)	S1—C8—H8B	109.5
C3—C2—C1	121.0 (2)	H8A—C8—H8B	108.1
C3—C2—H2	119.5	N1—C9—C10	111.90 (13)
C1—C2—H2	119.5	N1—C9—H9A	109.2
C2—C3—C4	119.9 (2)	C10—C9—H9A	109.2
C2—C3—H3	120.0	N1—C9—H9B	109.2
C4—C3—H3	120.0	C10—C9—H9B	109.2
C3—C4—C5	120.3 (2)	H9A—C9—H9B	107.9
C3—C4—H4	119.9	O1—C10—N2	122.83 (15)
C5—C4—H4	119.9	O1—C10—C9	123.14 (14)
C4—C5—C6	120.8 (2)	N2—C10—C9	113.99 (13)
C4—C5—H5	119.6	C7—N1—C6	124.21 (13)
C6—C5—H5	119.6	C7—N1—C9	115.53 (14)
C5—C6—C1	118.46 (16)	C6—N1—C9	120.12 (13)
C5—C6—N1	121.03 (15)	C10—N2—N3	122.91 (15)
C1—C6—N1	120.49 (15)	C10—N2—H1N	118.4 (13)
O2—C7—N1	121.60 (17)	N3—N2—H1N	117.0 (14)
O2—C7—C8	120.86 (17)	N2—N3—H2N	105.3 (19)
N1—C7—C8	117.53 (15)	N2—N3—H3N	105.6 (16)
C7—C8—S1	110.54 (14)	H2N—N3—H3N	112 (2)
C7—C8—H8A	109.5	C1—S1—C8	95.78 (9)
			
C6—C1—C2—C3	1.6 (3)	O2—C7—N1—C6	−178.88 (18)
S1—C1—C2—C3	−177.5 (2)	C8—C7—N1—C6	−0.2 (2)
C1—C2—C3—C4	−0.8 (4)	O2—C7—N1—C9	−3.3 (2)
C2—C3—C4—C5	−0.7 (4)	C8—C7—N1—C9	175.45 (16)
C3—C4—C5—C6	1.2 (3)	C5—C6—N1—C7	155.73 (17)
C4—C5—C6—C1	−0.3 (3)	C1—C6—N1—C7	−25.7 (2)
C4—C5—C6—N1	178.29 (17)	C5—C6—N1—C9	−19.7 (2)
C2—C1—C6—C5	−1.1 (3)	C1—C6—N1—C9	158.89 (15)
S1—C1—C6—C5	178.02 (14)	C10—C9—N1—C7	−76.87 (18)
C2—C1—C6—N1	−179.71 (19)	C10—C9—N1—C6	98.95 (17)
S1—C1—C6—N1	−0.6 (2)	O1—C10—N2—N3	4.2 (3)
O2—C7—C8—S1	−135.26 (18)	C9—C10—N2—N3	−178.15 (17)
N1—C7—C8—S1	46.0 (2)	C2—C1—S1—C8	−143.10 (19)
N1—C9—C10—O1	−26.9 (2)	C6—C1—S1—C8	37.80 (16)
N1—C9—C10—N2	155.47 (14)	C7—C8—S1—C1	−58.15 (15)

### Hydrogen-bond geometry (Å, °)

**Table:**

*D*—H···*A*	*D*—H	H···*A*	*D*···*A*	*D*—H···*A*
N3—H3N···O2^i^	0.96 (3)	2.35 (3)	3.299 (3)	171 (2)
N2—H1N···O1^ii^	0.87 (2)	2.07 (2)	2.935 (2)	175.5 (18)
C3—H3···O2^iii^	0.93	2.48	3.406 (3)	174
C8—H8B···O1^iv^	0.97	2.57	3.442 (2)	150

Symmetry codes: (i) *x*, *y*+1, *z*; (ii) *x*, −*y*+1, *z*+1/2; (iii) *x*+1/2, −*y*+1/2, *z*−1/2; (iv) *x*, *y*−1, *z*.
